# Immunogenicity of anthrax recombinant peptides and killed spores in goats and protective efficacy of immune sera in A/J mouse model

**DOI:** 10.1038/s41598-018-35382-8

**Published:** 2018-11-16

**Authors:** Okechukwu C. Ndumnego, Susanne M. Koehler, Jannie E. Crafford, Wolfgang Beyer, Henriette van Heerden

**Affiliations:** 1 Department of Veterinary Tropical Diseases, University of Pretoria, Onderstepoort, South Africa; 2 Institute of Animal Science, Department of Livestock Infectiology and Environmental Hygiene, University of Hohenheim, Stuttgart, Germany; 3 Present Address: Africa Health Research Institute, Durban, South Africa; 4 Present Address: Robert Koch Institute, Berlin, Germany

## Abstract

Anthrax is primarily recognized as an affliction of herbivores with incubation period ranging from three to five days post-infection. Currently, the Sterne live-spore vaccine is the only vaccine approved for control of the disease in susceptible animals. While largely effective, the Sterne vaccine has several problems including adverse reactions in sensitive species, ineffectiveness in active outbreaks and incompatibility with antibiotics. These can be surmounted with the advent of recombinant peptides (non-living) next generation vaccines. The candidate vaccine antigens comprised of recombinant protective antigen (PA), spore-specific antigen (bacillus collagen-like protein of anthracis, BclA) and formaldehyde inactivated spores (FIS). Presently, little information exists on the protectivity of these novel vaccine candidates in susceptible ruminants. Thus, this study sought to assess the immunogenicity of these vaccine candidates in goats and evaluate their protectivity using an *in vivo* mouse model. Goats receiving a combination of PA, BclA and FIS yielded the highest antibody and toxin neutralizing titres compared to recombinant peptides alone. This was also reflected in the passive immunization experiment whereby mice receiving immune sera from goats vaccinated with the antigen combination had higher survival post-challenge. In conclusion, the current data indicate promising potential for further development of non-living anthrax vaccines in ruminants.

## Introduction

Anthrax, a disease widely recognized as a primary disease of ruminants^1^, is caused by the Gram-positive, aerobic, spore-forming bacterium *Bacillus anthracis*. In nature the disease is mainly transmitted to susceptible herbivores by viable dormant spores present in contaminated foliage and soils. It progresses as a hyperacute infection in ruminants following an incubation period of three-five days^2^. The main virulence factors are encoded by two plasmids; pXO1, which codes for the three toxin components of protective antigen (PA), lethal factor (LF) and edema factor (EF)^3–5^ whereas pXO2 encodes the anti-phagocytic poly-ɣ-D-glutamic acid capsule (PDGA)^6^. The full virulence of *B. anthracis* depends on the existence of both plasmids. PA bonds with LF, forming lethal toxin (LT), a zinc metalloprotease that inactivates several mitogen-activated protein kinase kinases (MAPKK) leading to impairment and death of susceptible macrophages and other immunocompetent cells^7,8^. Edema toxin (ET) is a calmodulin dependent adenylate cyclase formed by the combination of PA to EF and which catalyses the production of cyclic AMP from the host ATP leading to the disruption of fluidic homeostasis in the host cells^9,10^.

The poorly immunogenic PDGA facilitates the dissemination of *B. anthracis* in the body of infected animals^11^. Masking of anthrax bacilli by the PDGA capsule enables it to avoid immune surveillance mechanisms and to proliferate systemically once inside the circulatory system^12,13^. Research has linked the PDGA with LT in the blood of experimentally infected animals^14^ and this was shown to significantly enhance the deleterious effects of LT in mice^15^.

The Sterne live spore vaccine is currently the only vaccine of choice for the control of anthrax in domestic animals. It is an attenuated pXO1+, pXO2− strain (34F2)^16^ known to induce good levels of immunity without clinical signs of the disease. However, some of the limitations of this vaccine include possible adverse reactions in some sensitive species^17–19^, short term protection^20^, ineffectiveness in active outbreaks and incompatibility with antibiotics^21,22^. Thus, the development of an alternative vaccine that can be produced quickly in the face of an anthrax outbreak, safe to administer and compatible with antibiotics is invaluable.

Induction of antibodies against PA is the main immune response following vaccination of animals with the Sterne live spore vaccine^23–25^. The anti-PA antibodies prevent the development of lethal intoxication and is vital for protection against germinating virulent anthrax bacilli^26^. Adding other anthrax antigens to PA-based vaccine candidates has been reported to improve the protection afforded to laboratory animals challenged with virulent anthrax spores^27^. An ideal non-living recombinant protein-based anthrax vaccine should be able to induce broad spectrum immunity targeting both toxaemia and bacteraemia. In the current study, an acellular vaccine formulation comprising of recombinant PA (rPA) and two other antigens; Bacillus collagen-like protein of anthracis (rBclA) and formaldehyde inactivated spores (FIS) were administered in a goat model and the resulting immune response and protection evaluated. BclA is an immunodominant glycoprotein found on the surface of the *B. anthracis* exosporium^28,29^, Previously, the addition of BclA to PA constructs had offered superior protection against virulent challenge in mice^30,31^ while FIS with PA-based vaccines significantly augmented the protection afforded to mice and guinea pigs^32,33^. We assessed the antibody response to rPA, rBclA, FIS and a lipopeptide adjuvant following vaccination in goats. The adjuvant comprised of *N*-palmitoyl-*S*-[2,3-bis(palmitoyloxy)-(2*R*, *S*)-propyl]-(*R*)-cysteinyl-seryl-(lysyl)_3_-lysine (Pam_3_Cys-SK_4_), a potent TLR2/1 activator admixed to Pam_3_Cys conjugated to FISEAIIHVLHSRHPG, a T-helper cell epitope from the sperm whale myoglobin^34–36^. We have previously shown that 70% of NMRI mice vaccinated with a combination of PA, BclA and lipopeptide adjuvant were protected against virulent challenge with *B. anthracis* Ames strain spores^37^. The adjuvant is well-defined, superior to conventional preparations and shows no untoward effects in animals^38^.

## Results

### Humoral immune response to non-living anthrax antigens in goats

**Figure 1:**
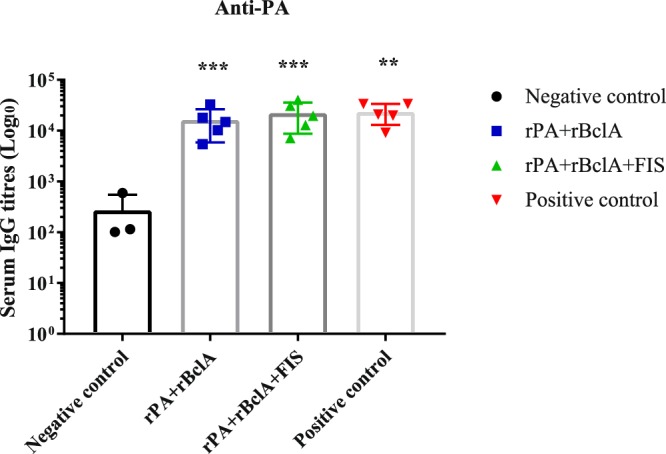
Individual anti- protective antigen (PA) IgG titres in goats (with mean bars and SD). The animals were either vaccinated thrice with rPA+rBclA (n =5) and rPA+rBclA+FIS (n =5) on weeks 0, 3 and 6 (sera collected for analyses on week 10) in combination with lipopeptide adjuvant or twice with Sterne live spore (n = 5) on weeks 0 and 12 (sera collected for analyses on week 17). The naïve controls (n = 3) received the vaccine diluent. IgG titres of each group were compared to the respective pre-immune titres (**P < 0.005; ***P < 0.0005). rPA; Recombinant protective antigen 83rBclA; Recombinant bacillus collagen-like protein of anthracisFIS; Formaldehyde inactivated spore.

Five goats each were vaccinated subcutaneously with rPA + rBclA+ lipopeptide adjuvant or rPA + rBclA + FIS+ lipopeptide adjuvant on weeks 0, 3 and 6. The jugular blood of each animal was sampled before each vaccination and again on week 10. Goats vaccinated twice with the Sterne live spore vaccine and vaccine diluent served as controls. Prepared sera were tested for IgG against PA, BclA, whole spores and lethal toxin neutralizing ability. Mean IgG titres against PA rose significantly after the first vaccination with rPA + rBclA + FIS+ lipopeptide adjuvant (*P* = 0.023) and increased after the second vaccination (Table 1). Significant changes in levels of anti-PA IgG were observed after the second vaccination with rPA + rBclA+ lipopeptide adjuvant. The highest increases in titres were observed after a second vaccination with either of the antigen combinations. A third vaccination only effected marginal changes in the recorded titres. The pre-challenge titres were equivalent with those observed following vaccinations with the Sterne live spore vaccine (Fig. 1).

**Table 1:** Antibody titres^ab^ (log_10)_ of goats vaccinated on weeks 0, 3 and 6 with recombinant proteins (rPA + rBclA) and lipopeptide adjuvant or recombinant proteins, inactivated spores and lipopeptide adjuvant (rPA + rBclA + FIS).

Vaccine group		Pre-vaccination	Week 3	Week 6	Week 10
**rPA** + **rBclA**	Anti-PA IgG	2.41 ± 0.37	2.87 ± 0.55	4.44 ± 0.23***	4.14 ± 0.36***
	Anti-BclA IgG	2.42 ± 0.30	2.85 ± 0.22*	3.28 ± 0.19**	3.19 ± 0.24***
	Anti-spore IgG	0.71 ± 1.20	0.35 ± 0.98	1.07 ± 1.22	0.68 ± 1.16
	TNA^c^	n.d.	0.37 ± 1.04	2.95 ± 0.25***	2.79 ± 0.54***
**rPA** + **rBclA** + **FIS**	Anti-PA IgG	2.09 ± 0.22	2.59 ± 0.33*	3.91 ± 0.38***	4.27 ± 0.37***
	Anti-BclA IgG	2.06 ± 0.14	2.51 ± 0.23*	3.10 ± 0.35**	3.19 ± 0.48**
	Anti-spore IgG	1.16 ± 1.31	3.05 ± 0.42*	3.32 ± 0.23*	2.99 ± 0.18*
	TNA^c^	n.d.	0.34 ± 0.95	2.61 ± 0.33***	2.92 ± 0.47***

^a^Mean log_10_ titres ± 95% confidence interval.

^b^Titres were compared to the respective pre-vaccination titres (*P < 0.05; **P < 0.005; ***P < 0.0005).

^c^Lethal toxin neutralization titres

rPA; Recombinant protective antigen 83.

rBclA; Recombinant bacillus collagen-like protein of anthracis.

FIS; Formaldehyde inactivated spore.

n.d; Not detected.

**Figure 2:**
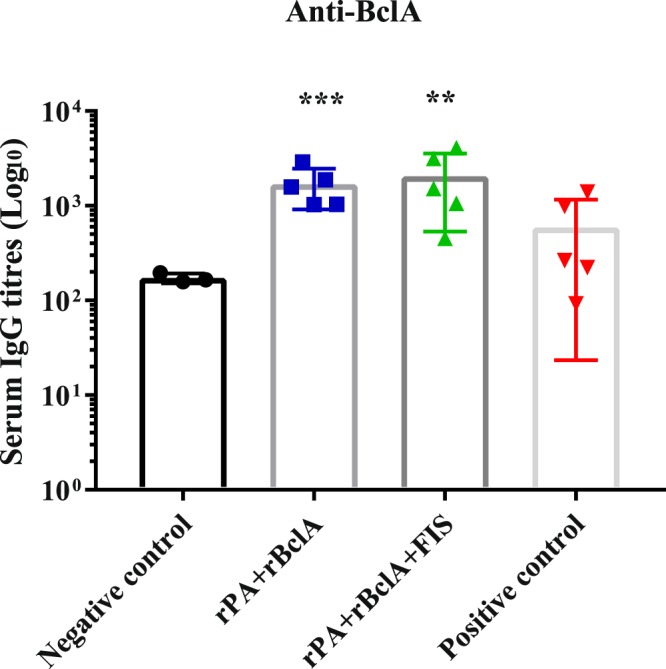
Individual anti-BclA IgG titres in goats (with mean bars and SD). The animals were either vaccinated thrice with rPA+rBclA (n = 5) and rPA+rBclA+FIS (n = 5) together with lipopeptide adjuvant on weeks 0, 3 and 6 (sera collected for analyses on week 10) or twice with Sterne live spore (n = 5) on weeks 0 and 12 (sera collected for analyses on week 17). The naïve controls (n = 5) received the vaccine diluent. IgG titres of each group were compared to the respective pre-immune titres (*P < 0.05; **P < 0.005; ***P < 0.0005).rPA; Recombinant protective antigen 83rBclA; Recombinant bacillus collagen-like protein of anthracisFIS; Formaldehyde inactivated spore.

**Figure 3:**
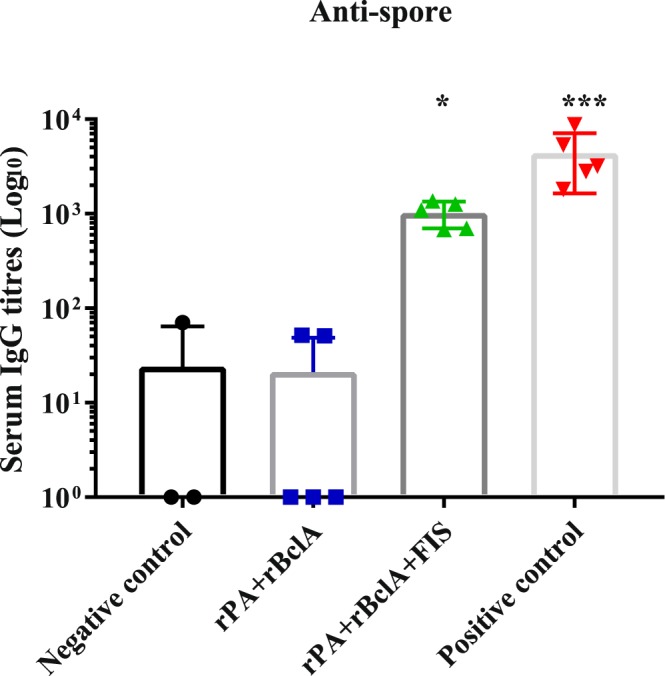
Individual anti-spore IgG titres in goats (with mean bars and SD). The animals were either vaccinated thrice with rPA+rBclA (n = 5) and rPA+rBclA+FIS (n = 5) together with lipopeptide adjuvant on weeks 0, 3 and 6 (sera collected for analyses on week 10) or twice with Sterne live spore (n = 5) on weeks 0 and 12 (sera collected for analyses on week 17). The naïve controls (n = 5) received the vaccine diluent. IgG titres of each group were compared to the respective pre-immune titres (*P < 0.05; **P < 0.005; ***P < 0.0005).rPA; Recombinant protective antigen 83rBclA; Recombinant bacillus collagen-like protein of anthracisFIS; Formaldehyde inactivated spore.

A significant increase of anti-BclA IgG was observed in goats vaccinated with rPA + rBclA and rPA + rBclA + FIS (*P* ≤ 0.031) after the first vaccination. Generally, the anti-BclA titres were a log lower than anti-PA titres after the second or third vaccination (Table 1). These antibodies were insignificant in the Sterne-vaccinated goats throughout the trials (Fig. 2). Conversely, the anti-spore IgG response in the Sterne-vaccinated group was higher when compared with any of the groups vaccinated with the non-living vaccine candidates (*P* ≤ 0.002), though also significantly elevated in the FIS-containing group (Fig. 3). While production of anti-spore IgG was evident following the first vaccination with rPA + rBclA + FIS, none was observed throughout the study in the rPA + rBclA-vaccinated animals (Table 1 and Fig. 3).

**Figure 4:**
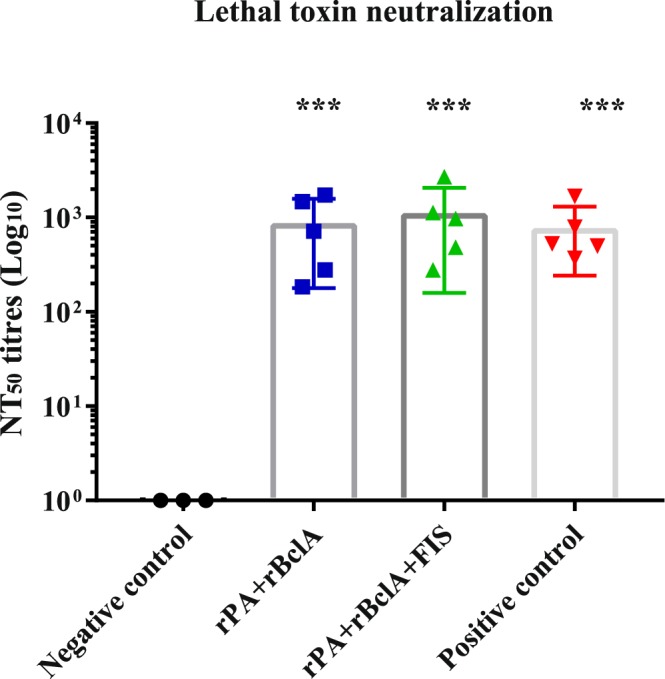
Individual anthrax lethal toxin neutralization titres in goats (with mean bars and SD). The animals were either vaccinated thrice with rPA+rBclA (n = 5) and rPA+rBclA+FIS (n = 5) with lipopeptide adjuvant on weeks 0, 3 and 6 (sera collected for analyses on week 10) or twice with Sterne live spore (n = 5) on weeks 0 and 12 (sera collected for analyses on week 17). The naïve controls (n = 3) received the vaccine diluent. IgG titres of each group were compared to the respective pre-immune titres (***P < 0.0005). rPA; Recombinant protective antigen 83rBclA; Recombinant bacillus collagen-like protein of anthracisFIS; Formaldehyde inactivated spore.

The development of significant lethal toxin neutralizing antibodies was only seen after the second vaccination with either of the non-living vaccine combinations (Table 1). Thereafter it followed the pattern as seen with anti-PA IgG titres but at a lower level. There was no difference in the TNA titres after the second or third vaccination (*P* ≥ 0.152). Following the stabilization of the neutralizing antibody levels after the second vaccination, the titres remained steady till the end of the experiment and were equivalent to the Sterne-vaccinated controls (Fig. 4).

### Protection conferred on A/J mice by caprine immune sera

**Figure 5:**
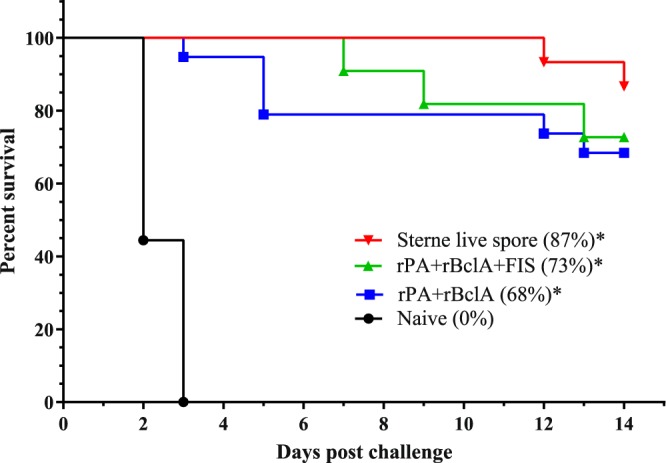
Passive protection of A/J mice following in vivo transfer of immune sera from goats and lethal challenge with ~1.92 × 105 Sterne 34F2 spores. The rPA+rBclA+FIS (n = 11) and rPA+rBclA (n = 19) groups with lipopeptide adjuvant received sera from goats vaccinated thrice with the respective vaccine candidates (also see Table 1). The Sterne live spore group (n = 15) sera were collected from goats vaccinated twice with Sterne strain spores. Naive (n = 9) sera were from goats injected with vaccine diluent (saline). *** denotes significantly increased survival as compared to the negative control (P < 0.001). There was no difference between the survival times of the Sterne live spore, rPA+rBclA+FIS and rPA+rBclA vaccinated groups (P ≥ 0.189). rPA; Recombinant protective antigen 83rBclA; Recombinant bacillus collagen-like protein of anthracisFIS; Formaldehyde inactivated spore.

To assess the protective efficacy of the immune response generated by vaccination of goats with anthrax recombinant proteins and inactivated spores, a passive *in vivo* mouse protection experiment was carried out after adoptive caprine sera transfer. Following lethal challenge with ~1.92 × 10^5^ Sterne 34F2 *B. anthracis* spores, sera from naïve goats failed to protect the susceptible mice with all the challenged mice dying within 3 days (Fig. 5). Sera from rPA + rBclA-vaccinated goats protected 68% of the challenged mice and 73% were protected by rPA + rBclA + FIS immune sera. Sera from the Sterne vaccinates protected 87% of challenged A/J mice. Analysis of the survival data from the groups showed no difference in survival between the rPA + rBclA, rPA + rBclA + FIS and Sterne vaccinated groups (*P* ≥ 0.189). On the other hand, these groups showed increased survival times compared to the naïve (negative) controls (*P* < 0.001).

To ascertain if there is any association between the measured antibody titres and survival against anthrax in the A/J mice, a Pearson’s correlation study was performed. This revealed a strong positive relationship between anti-PA, anti-spore and toxin neutralizing antibody titres and survival in the mice (Table 2). There was no significant correlative association between anti-BclA IgG titres and survival in the challenged mice.

**Table 2:** Pearson’s correlation analysis of relationship between survival times of passively challenged A/J mice and caprine sera antibody titres.

		Anti-PA IgG	Anti-spore IgG	TNA^a^	Anti-BclA IgG
Survival	Pearson Correlation	0.460**	0.357**	0.336*	0.251
	Significance (2-tailed)	<0.001	0.008	0.013	0.067

^a^Lethal toxin neutralization titres.

^*^Correlation is significant at the 0.05 level (2-tailed).

**Correlation is significant at the 0.01 level (2-tailed).

## Discussion

In addition to performing the trials in this study under more controlled conditions, evaluation of the protective capacity of immune sera was performed using an *in vivo* mouse challenge model. This model has been used previously albeit in homogenous mouse sera transfer^39^ or adoptive heterogenous immune sera transfer from Sterne live spore vaccinates^20^. To the best of our knowledge, this study represents the first attempt to assess a recombinant anthrax vaccine candidate in a ruminant model using a passive mice protection model. Immune sera from goats vaccinated with combinations of rPa + rBclA + FIS and rPA + rBclA were able to protect 73% and 68% of A/J mice from lethal anthrax challenge respectively. Immune titres peaked following a single booster vaccination with the non-living vaccine candidates, indicating the expendability of a second booster vaccination.

Anthrax is chiefly a disease of herbivorous animals with ruminants being most susceptible^1,2^. The disease is largely controlled using the Sterne live spore vaccine which is an unencapsulated but toxinogenic strain^40,41^. The vaccine retains some residual virulence, though attenuated and has limited effectiveness in the face of an active anthrax outbreak^22,42^. With the advent of multicomponent recombinant anthrax antigens capable of stimulating broad spectrum immune response in vaccinates, questions have risen about the potential usefulness of such vaccine candidates in the control of the disease in livestock. While the antigens of *B. anthracis* rPA, rBclA and FIS, alone or in combination are recognized for their protective efficacy from many laboratory rodent studies^27^, it remains to be elucidated if these antigens will elicit a similar response in a ruminant model of anthrax infection. Recently, we tested a combination of rPA and rBclA with or without FIS in a mixed breed of farm goats, under field conditions^43^. In spite of low lethal toxin neutralizing titres, measured at point of lethal challenge, 80% of goats receiving rPA, rBclA and FIS were protected from direct lethal challenge with *B. anthracis* spores^43^.

PA is responsible for the production of toxin neutralizing antibodies which is vital for protection against anthrax infections^26,44^. It is the principal immunogen of the licensed human vaccine^45^ and forms the primary component of numerous recombinant anthrax candidate vaccines. The measure of anti-PA and toxin neutralizing antibodies peaked after a second vaccination with either of rPA + rBclA or rPA + rBclA + FIS and lipopeptide adjuvant. This was also replicated in the production of anti-BclA and anti-spore (rPA + rBclA + FIS only) antibodies. The results indicated that these recombinant vaccines were able to induce strong immune responses comparable to the Sterne live spore vaccinated controls. The humoral response to BclA in the Sterne vaccinated controls was poor, which was noted previously^24^. Previous studies in mice^37^ and rabbits (unpublished data) had recorded antibody titres against the BclA used in this study. Notwithstanding, almost 90% of challenged mice were protected by Sterne vaccinates sera implying the insignificant role of anti-BclA antibodies in protection against anthrax in these animals, a fact also observed in the direct challenge of Sterne vaccinated goats^24^. The addition of FIS to the rPA + rBclA vaccine combination increased survival (not significant) by 5% (Fig. 5). In an earlier study using similar vaccine candidates, 50% of goats vaccinated with rPA + rBclA were protected against direct virulent *B. anthracis* spores challenge compared to 80% survival observed in rPA + rBclA + FIS-vaccinated goats^43^. Though not significant, more mice were protected by the FIS, BclA and PA vaccinates’ sera compared to PA and BclA alone (Fig. 5). A correlative study of the association between antibody titres and survival reinforced this assumption with anti-PA, anti-spore and toxin neutralization titres showing positive correlations (Table 3). A possible optimal combination of possible vaccine candidates (for future trials) based on our results will be a PA and FIS vaccine with the addition of an adjuvant. Not only will this combination be less complex, but also potentially easier to mass produce. A similar PA and FIS vaccine combination was shown to afford better protection in mice and guinea pig models previously^32,33^.

**Table 3:** Vaccine group designations, dosage and schedule.

Group (x number of vaccinations)	Number of goats	Dosage (Subcutaneous)	Vaccination, weeks	Number of Mice (*in vivo* challenge)
rPA + rBclA × 3	5	rPA = 75 µg/dose rBclA = 75 µg/dose Lipopeptide adjuvant = 500 µg/dose	0, 3, 6	19^†^
rPA + rBclA + FIS × 3	5 (3)*	rPA = 75 µg/dose rBclA = 75 µg/dose FIS = 10^8^ spores/dose Lipopeptide adjuvant = 500 µg/dose	0, 3, 6	11^†^
Negative (naive) × 1	3	1 mL of vaccine diluent	0	9 (3 mice tested/goat)
Positive × 2	5	Goats vaccinated twice with 1 mL Sterne live spore vaccine	0, 12	15 (3 mice tested/goat)

^†^Some mice reacted adversely from *in vivo* goat serum transfer and either died or were euthanized.

*Sera of three goats assessed by *in vivo* challenge due to adverse reactions in mice.

rPA; Recombinant protective antigen 83.

rBclA; Recombinant bacillus collagen-like protein of anthracis.

FIS; Formaldehyde inactivated spore.

Except for the anti-spore response, there was little difference in the humoral response of the recombinant vaccinated animals. The *in vivo* A/J mouse protection model evaluates the level of protection afforded by induced humoral antibodies following vaccination^20,39,46^. In this model, the toxin-neutralizing efficacy of these antibodies in protection of the susceptible A/J mice against lethal challenge with *B. anthracis* Sterne strain (toxin+ capsule−)^47^ is critical. The ~70% protection observed following vaccination with rPA + rBclA and rPA + rBclA + FIS in this study demonstrates the potential protective capacity in the donor animal. The lipopeptide adjuvant used in this study, Pam_3_Cys-SK_4_ represents a highly efficient immunoadjuvant used in peptide/protein vaccination (Mittenbuhler *et al*. 2003). Pam_3_Cys-SK_4_ has been reported severally to enhance the humoral immune response to antigens in various species^38,48–51^. Co-administration of the adjuvant with the recombinant proteins was well tolerated by the goat hosts throughout the trials. The choice of 500 µl as the volume of passively transferred immune sera was based on previous studies^20,39^ and after consultations with PCB Turnbull. The adverse reactions in some of the mice following intraperitoneal serum transfer were unexpected. The post mortem reports on the affected mice indicated possible anaphylactic reactions (serum sickness) due to the serum infusion (not shown). This may have been dependent on the individual immune status of the mice affected as repetitions with the same volume of serum did not result in further deaths. On hindsight, the use of affinity-purified antibodies from immune sera could have prevented these reactions.

In summary, our study reveals the potential of a non-living anthrax vaccine in inducing a protective immune response in vaccinated goats. Results indicate the protective capacity induced in caprine sera following vaccination with either rPA + rBclA or rPA + rBclA + FIS in combination with a lipopeptide adjuvant.

## Materials and Methods

### Preparation and purification of recombinant proteins and *Bacillus anthracis* spores

*Escherichia coli* BL21-CodonPlus-RIL cells (Stratagen, LaJolla, USA) retaining the plasmid pREP4 (Qiagen, Venlo, Netherlands) and pQE-30 (Qiagen, Venlo, Netherlands) that encodes either rPA83 or rBclA were cultured and refined as previously described^24,52^. Proteins used for ELISA received no further treatment whilst proteins for vaccination were tested for endotoxin using the Limulus Amoebocyte Lysate Endochrome-K test kit (Charles River, Wilmington, USA) as described by the manufacturer. Endotoxin removal was carried out via EndoTrap blue endotoxin removal system (Hyglos, Bernried, Germany).

The FIS suspension from Sterne vaccine strain (34F2) done as previously^53^ with some modifications. Spores were incubated at 37 °C overnight in PBS at concentrations containing at least 10^9^ spores/mL and formaldehyde at a final concentration of 4% formalin. Subsequently, after pelleting at 4000 × *g* for 15 min (room temperature), the spores were washed four times with PBS in 0.1% gelatine. This was followed by resuspension in PBS and storage at −80 °C. Aliquots of the FIS suspension was tested for sterilization by streaking on blood agar after treatment with histidine (to neutralize any remnant formalin).

*B. anthracis* 34F2 Sterne spores (batch no. 86) utilized for goat immunization and *in vivo* mouse challenge were sourced from Onderstepoort Biological Products (OBP, Onderstepoort, South Africa). The spores were prepared by the dilution and centrifugation of the spores with sterile PBS/glycerin (0.1%) solution at 3000 × *g* for 15 min at 4 °C. The final spore suspension was treated at 65 °C for 30 min in a water bath before counting and storage at 4 °C in sterile PBS/glycerine solution.

### Immunization experiments and passive protection tests

Goats were screened for PA-reactive antibodies using the conventional PA ELISA. Following arrival and acclimatization at the OBP experimental animal facility, the goats were randomly allocated to designated vaccine groups and immunized as indicated in Table 3. A/J mice (Jackson Laboratories, Bar Harbor, USA) were procured for the *in vivo* challenge study performed at the University of Pretoria Biomedical Research Centre (UPBRC), South Africa. The A/J mouse strain is deficient in the C5 complement component which renders it vulnerable to infection by vaccine-strain Sterne spores^54,55^. These mice develop lethal systemic anthrax following infection with Sterne spores in the absence of protective antibodies^20,25,39^. Experiments with animals were conducted in accordance with ethical principles and guidelines provided by the animal use and care committees of the UPBRC, OBP and University of Pretoria respectively (Protocol number V065/12). Section 20, Act 35 of 1984 permission was granted by the Directorate of Animal Health, Department of Agriculture, Forestry and Fisheries, South Africa (registration number 12/11/1/1/6).

For the passive protection tests, sera (500 µl) from vaccinated goats and controls (naive and hyperimmune goats) were injected intraperitoneally into naïve A/J mice. Following the transfer of caprine sera into the A/J mice, lethal challenge was performed 24 h later with *B. anthracis* Sterne strain. In the experimental design, a total of 5 mice per goat serum (for the protein-vaccinated groups) and 3 mice per serum [for the Sterne-vaccinated (positive) and naïve (negative) controls] were used. However, due to the unexpected adverse reactions shown by some of the mice following the adoptive transfer of sera and subsequent exclusion from the trials, the challenge experiment was performed with reduced mice number for some of the serum samples (Table 3). In rPA + rBclA group, two goat immune sera were tested with five mice each while sera from three goats were assessed with four, three and two mice respectively (19 mice in total). Two caprine sera were assessed with five mice each in rPA + rBclA + FIS group and a serum sample was tested with one mouse (three sera samples were assessed in this group, 11 in total). Consequently, the challenge and survival data were pooled for each vaccine group.

### Measurement of serum IgG titres

Sera were analysed for specific immunoglobulins using ELISA as previously described by^52^ with some modifications. Individual wells of 96-well microtitre plates (Nunc immunoplate Maxisorp, Germany) were coated with 0.5 µg of antigen (or 10^7^ FIS) in bicarbonate buffer and incubated overnight at 4 °C. The plates were washed twice with PBS containing 0.05% Tween (PBST) using a Biorad PW40 washer (Mamesla-Coquette, France) and blocked in 200 µL PBST containing 5% skimmed milk powder (PBSTM) and incubated for 1 h at room temperature (RT). The plates were washed twice, and the test sera and controls diluted in PBSTM. A two-fold dilution (starting dilution; 1:50) was made across the plates in duplicates and incubated for 30 min on a rotatory shaker (Titretek® flow labs, UK). Following incubation, the plates were washed 5 times and 100 µL of horseradish peroxidase-conjugated rabbit anti-goat IgG (Invitrogen, Camarillo, USA) diluted to 1:4000 in PBSTM was added to every well and incubated for 30 min on the plate shaker. After washing 5 times, the plates were developed with 2,2′ azino bis (3-ethylbenzthiazoline-6-sulfonic acid) diammonium salt (Sigma, Germany) and absorbance readings taken at 405 nm using a Biotek powerwave XS2 reader (Winooski, USA). Endpoint titres of individual serum were defined as the reciprocal of the highest serum dilution giving an optical density of 0.1. Titres of <50 were ascribed an arbitrary value of 0.

### Toxin neutralization assay (TNA)

An *in vitro* toxin neutralizing assay (TNA) was carried out with J774A.1 mouse macrophage cell line (ECACC cat no 91051511) as done previously with modifications^24,56^. The 96-well flat-bottomed tissue culture plates (Greiner bio one, Germany) seeded with 80 000 macrophages per well in Dulbecco’s modified eagle media (DMEM) and 10% foetal calf serum (FCS) were incubated overnight at 37 °C and 5% CO_2_. Test sera were diluted 2-fold (starting dilution; 1:50) in culture medium with PA and LF (List Biological Laboratories Inc., Campbell, USA) at concentrations of 500 ng/mL and 400 ng/mL respectively. The serum/lethal toxin mixture was incubated for 1 h at 37 °C and 5% CO2 before addition to overnight-cultured cells and incubation for 3 h. Every serum sample was tested in duplicates. Subsequently, addition of 25 µL of 5 mg/mL MTT (3-(4,5 dimethylthiazol-2-yl)-2,5-diphenyltetrazolium bromide; Invitrogen, USA) per well and incubation in the dark at 37 °C and 5% CO_2_ was performed. Two hours after incubation, cells were lysed with pre-warmed (37 °C) acidified isopropyl alcohol (90% isopropyl alcohol, 0.5% SDS, 25 mM HCl) by vigorously pipetting up and down to solubilize the formazan dye.

Finally, plates were rested for 5 min and the absorbance read at 540 nm with a Biotek power wave XS2 reader. Each assay included a single dilution series of positive control serum from a goat hyper-immunized with the Sterne live spore vaccine serving as a positive control and for assay reproducibility. Three wells in each assay receiving LT served as blanks, another triplicate of wells (with cells) received only LT as toxin control while only culture media was placed in two wells (medium control). The neutralization of each serum sample was calculated using the formula:

$$N{T}_{{\rm{50}}}\,=(sample-toxin\,control)\div(medium\,control-toxin\,control)\times 100$$

Neutralization titres (NT_50_) were expressed as the reciprocal of the highest serum dilution at which the macrophage survival yielded 50% and obtained using the Gen5 data analysis software (Biotek Instruments, USA).

### Statistics

For determination of ELISA and TNA titres, 4-parametre logistic regression curves were generated from serial dilution data using the Gen 5 data analysis software (Biotek Instruments, Winooski, USA). Data obtained were log-transformed and variance equality were tested with the Levene’s tests (IBM SPSS Statistics 23). Antibody titre differences between groups at particular time points were analysed using unpaired student *t*-test, with a two-tailed *P*-value. Baseline and pre-challenge ELISA and TNA data within vaccine groups were compared using a paired Student’s *t* test. Kaplan-Meier (product limit estimation) plots were used to compute the mean survival times of challenged mice (pooled per treatment group). Survival curves were compared between each treatment group and controls using the Mantel-Cox (Log Rank) test. The strength of association between the survival time following lethal challenge and specific immune titres was measured using the Pearson’s correlation coefficient. Results having a *p* value of ≤ 0.05 were considered statistically significance.

## Data Availability

The datasets generated and analysed during the current study are available from the corresponding author on reasonable request.
